# Experimental Infection and Transmission of SARS-CoV-2 Delta and Omicron Variants among Beagle Dogs

**DOI:** 10.3201/eid2904.221727

**Published:** 2023-04

**Authors:** Kwang-Soo Lyoo, Hanbyeul Lee, Sung-Geun Lee, Minjoo Yeom, Joo-Yeon Lee, Kyung-Chang Kim, Jeong-Sun Yang, Daesub Song

**Affiliations:** Jeonbuk National University, Iksan, South Korea (K-S. Lyoo, S.-G. Lee);; Seoul National University, Seoul, South Korea (H. Lee, M. Yeom, D. Song);; Korea Disease Control and Prevention Agency, Cheongju, South Korea (J.-Y. Lee, K.-C. Kim, J.-S. Yang)

**Keywords:** COVID-19, severe acute respiratory syndrome coronavirus-2, SARS-CoV-2, coronavirus disease, variants, viruses, dogs, respiratory infections, infections, disease transmission, Delta, Omicron, South Korea

## Abstract

We assessed susceptibility of dogs to SARS-COV-2 Delta and Omicron variants by experimentally inoculating beagle dogs. Moreover, we investigated transmissibility of the variants from infected to naive dogs. The dogs were susceptible to infection without clinical signs and transmitted both strains to other dogs through direct contact.

Since COVID-19 was first reported in China in late 2019 and quickly became a worldwide pandemic, zoonotic aspects of SARS-CoV-2 have raised public health concerns (*1*). The first reported case of SARS-CoV-2 infection in a companion animal was in Hong Kong (*2*). Pet dogs living with patients affected by COVID-19 shed low levels of SARS-CoV-2 and show seroconversion without any clinical signs (*2*). Multiple cases of SARS-CoV-2 transmission from humans to dogs have since been reported in several countries (*3*). In experimental infection studies, dogs inoculated with SARS-CoV-2 wild-type strain manifested no clinical signs or seroconversion, shed low titers, or had undetectable viral RNA (*4*,*5*). As companion animals, dogs commonly share living spaces with humans; therefore, more studies are needed to elucidate the susceptibility of dogs to SARS-CoV-2 infection. 

SARS-CoV-2 variants Delta, in late 2020, and Omicron, in 2021, emerged and quickly spread worldwide. Those variants have been characterized by more efficient human-to-human transmission than the wild-type strain (*6*). In this study, we assessed susceptibility of beagle dogs to SARS-CoV-2 Delta and Omicron variants and transmissibility of SARS-CoV-2 variants from infected to naive dogs. 

## The Study

We obtained SARS-CoV-2 Omicron (NCCP 43408, BA.1. lineage) and Delta (NCCP 43390, B.1.617.2 lineage) variants from the National Culture Collection for Pathogens of South Korea. We passaged the viruses twice in Vero E6 cells and titrated the virus stocks on Vero E6 cells using a 50% tissue culture infectious dose (TCID_50_) assay. This study was performed at the Animal Use Biosafety Level 3 facility of the Korea Zoonosis Research Institute. The Institutional Animal Care and Use Committee approved animal experiments (approval number JBNU 2022–033), and the Institutional Biosafety Committee of Jeonbuk National University approved experimental protocols requiring biosafety (approval no. JBNU2022–02–002).

We purchased 9 male beagle dogs, all 9 months of age, from Orient Bio Inc. (http://www.orient.co.kr). We intranasally inoculated 2 dogs with 10^6.0^ TCID_50_/mL SARS-CoV-2 Delta variant and 2 others with 10^6.0^ TCID_50_/mL SARS-CoV-2 Omicron variant (infected dogs). Twenty-four hours after infection, we housed 2 virus-naive dogs (transmission dogs) each with the infected dogs in separate large animal isolators, 1 for Delta-infected dogs and 1 for Omicron-infected dogs (total 4 dogs in each isolator, 2 infected and 2 naive). We assigned 1 naive dog as the noninfected negative control and kept it separate from the other dogs. We recorded body temperature and weight, and collected blood, nasal swabs, and rectal swabs the day of and 2, 4, 6, 8, and 10 days after infection for the infected dogs or days after cohousing began for the transmission dogs. All dogs were humanely killed 10 days after infection or cohousing, after which we collected lung tissue for histopathologic examination and viral load measurement. 

Using an Exigo C200 automatic analyzer (Boule Medical AB, https://boule.com), we tested for total protein, alkaline phosphatase, alanine aminotransferase, aspartate aminotransferase, lactate dehydrogenase, creatine kinase, creatinine, blood urea nitrogen, blood urea nitrogen/creatinine, and glucose. All results were within reference ranges except creatine kinase which, 4 days after infection or cohousing, was almost 8 times the upper limit of the normal range (0–200 U/L) in 1 infected dog (OI-1, 1,589 U/L) and 1 transmission dog (OT-1, 1,560 U/L) with Omicron variant (Appendix Figure). During the study, none of the dogs showed any clinical signs of illness, including weight loss or fever. 

**Figure 1:**
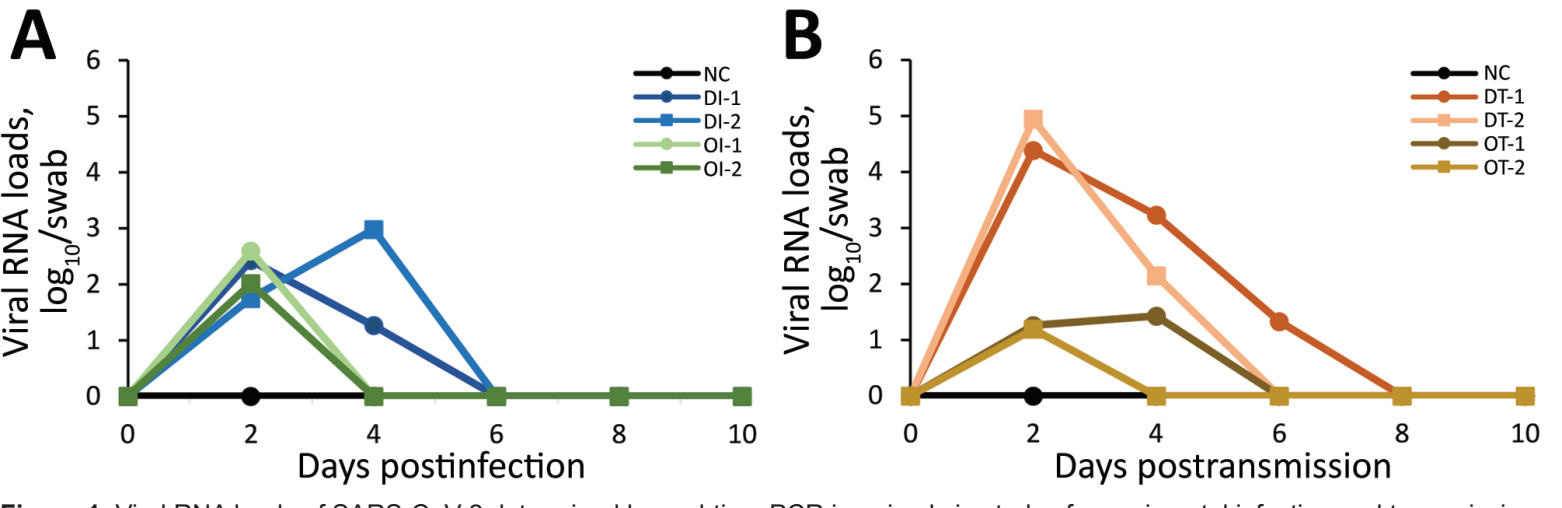
Viral RNA loads of SARS-CoV-2 determined by real-time PCR in animals in study of experimental infection and transmission of SARS-CoV-2 Delta and Omicron variants among beagle dogs. A) Viral loads in nasal swab samples from infected dogs. B) Viral loads in nasal swab samples from naive (transmission) dogs exposed to dogs infected with Delta or Omicron variants. NC, normal control; DI, delta variant infection; OI, omicron variant infection; DT, delta variant transmission; OT, omicron variant transmission.

To measure viral RNA loads of SARS-CoV-2 in lung tissues and swab samples, we used real-time quantitative PCR to detect the nucleocapsid gene of SARS-CoV-2 using TaqMan Fast Virus 1-Step Master Mix (Thermo Fisher Scientific, https://www.thermofisher.com), as described elsewhere (*7**,**8*). We cultivated viruses from all nasal swab samples using a TCID_50_ assay with Vero E6 cells. We did not detect viral RNA in lung tissue or rectal swab samples. However, the nasal swab samples taken from all dogs 2 days after infection with Delta or Omicron variants were positive for SARS-CoV-2 RNA; we also detected viral RNA in the swab samples taken from Delta-infected dogs 4 days after infection (Figure 1, panel A). In the SARS-CoV-2 transmission portion of the study, we detected viral RNA in nasal swab samples taken 2 days after transmission from dogs in the Delta and Omicron variant transmission groups; viral RNA loads from the 2 dogs in the Delta variant group (4.4 and 4.9 log_10_ genome copies/swab) were higher than the 2 in the Omicron variant group (1.2 and 1.3 log_10_ genome copies/swab) (Figure 1, panel B). Viral shedding from infected and transmission dogs was revealed by virus cultivation (Table). 

**Table:** Viral titers from nasal swab samples from animals in study of experimental infection and transmission of SARS-CoV-2 Delta and Omicron variants among beagle dogs

Dog group	Dog no.	Days after exposure					
		0	2	4	6	8	10
Control	NA	NA	NA	NA	NA	NA	NA
Infection							
Delta	1	NA	2.5	2.2	NA	NA	NA
	2	NA	3.2	2.7	NA	NA	NA
Omicron	1	NA	2.8	NA	NA	NA	NA
	2	NA	2.2	NA	NA	NA	NA
Transmission							
Delta	1	NA	3.7	2.8	NA	NA	NA
	2	NA	4.3	2.3	NA	NA	NA
Omicron	1	NA	1.8	2.0	NA	NA	NA
	2	NA	2.3	NA	NA	NA	NA

*Values are 50% tissue culture infectious dose/mL. NA, not applicable.

**Figure 2:**
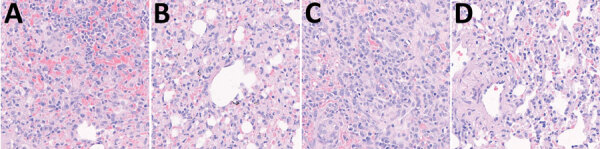
Pathologic changes in the lungs of dogs in study of experimental infection and transmission of SARS-CoV-2 Delta and Omicron variants among beagle dogs. A) Lung tissue from dog experimentally infected with Delta variant shows alveolar septa severely thickened by the infiltration of lymphocytes, macrophages, degenerate neutrophils, and karyorrhectic cellular debris. B) Lung tissue from naive (transmission) dog housed with Delta-infected dog shows alveolar septa thickened by the infiltration of numerous macrophages and lymphocytes, along with collagen accumulation. C) Lung tissue from dog experimentally infected with Omicron variant shows severe interstitial pneumonia and alveolar septal thickening due to the infiltration of lymphocytes, macrophages, and degenerate neutrophils. D) Lung tissue from naive (transmission) dog housed with Omicron-infected dog shows alveolar septa thickened by few macrophages, lymphocytes, and degenerate neutrophils. Original magnification ×400.

During necropsy at the end of the experiment, we found no gross lesions in any organ, but both infected and transmission dogs showed histopathologic changes in the lungs. The alveolar wall was locally thickened by infiltration of lymphocytes and monocytes, including macrophages, and proliferation of the alveolar epithelium (Figure 2). 

## Conclusions

Continuing emergence of SARS-CoV-2 variant strains presents a threat to public health and challenges the effectiveness of current vaccines. Delta and Omicron variants are more transmissible and resistant to neutralization than other strains in vaccinated persons (*6*). Although the SARS-CoV-2 pandemic has been driven mainly by human-to-human transmission, zoonotic viral infection from companion animals to humans has been reported frequently worldwide (*3*). To examine this dynamic, experimental infection studies have been performed using different animal species, including dogs (*3*,*5*).

For this study, we intranasally infected 9-month-old beagle dogs with SARS-CoV-2 Delta and Omicron variants; our results demonstrate that the dogs were susceptible to infection with and could transmit both strains to other dogs through direct contact. Despite no clinical signs, microscopic lesions were observed in the lungs of both infected and transmission dogs. Among the blood chemistry parameters, creatine kinase levels were markedly increased in Omicron-infected dogs. Creatine kinase is a marker of muscle damage, and elevated levels, such as those found among the Omicron-infected dogs, are associated with worse outcomes in respiratory patients infected with influenza viruses or SARS-CoV-2 (*9*,*10*). However, we could not exclude the possibility that creatine kinase could be elevated by muscle injury caused in an uncontrolled situation such as fighting among dogs instead of by SARS-CoV-2 infection. Therefore, further studies to clarify the role of blood chemistry parameters, such as creatine kinase, in animal models would be valuable. 

The higher infectivity of SARS-CoV-2 variants than the wild-type virus strain led us to perform this experimental infection and transmission study in dogs. SARS-CoV-2 variants shed from humans might infect and efficiently circulate among companion animals, such as dogs or cats (*11*,*12*). It has been hypothesized that those companion animals could subsequently zoonotically infect humans. This scenario raises concerns about possible spillover between humans and companion animals, which might require continuous surveillance to monitor SARS-CoV-2 variants in companion animals. In the future, successful vaccination strategies for companion animals might be effective as a public health intervention in protecting animals from infection and preventing zoonotic transmission from infected animals.

Appendix Additional information about SARS-CoV-2 Delta and Omicron variant infection and transmission among beagle dogs.
