# Supplementary material for: Experimental Infection and Transmission of SARS-CoV-2 Delta and Omicron Variants among Beagle Dogs
Source: Emerg Infect Dis. 2023 Apr;29(4):782–5. doi: 10.3201/eid2904.221727 (PMC10045707; doi:10.3201/eid2904.221727)
Supplement: Appendix — Additional information about SARS-CoV-2 Delta and Omicron variant infection and transmission among beagle dogs. [file 22-1727-Techapp-s1.pdf]

Article DOI: https://doi.org/10.3201/eid2904.221727

EID cannot ensure accessibility for supplementary materials supplied by authors. Readers who have difficulty accessing supplementary content should contact the authors for assistance.

Experimental Infection and Transmission of SARS-CoV-2 Delta and Omicron Variants among Beagle Dogs

Appendix

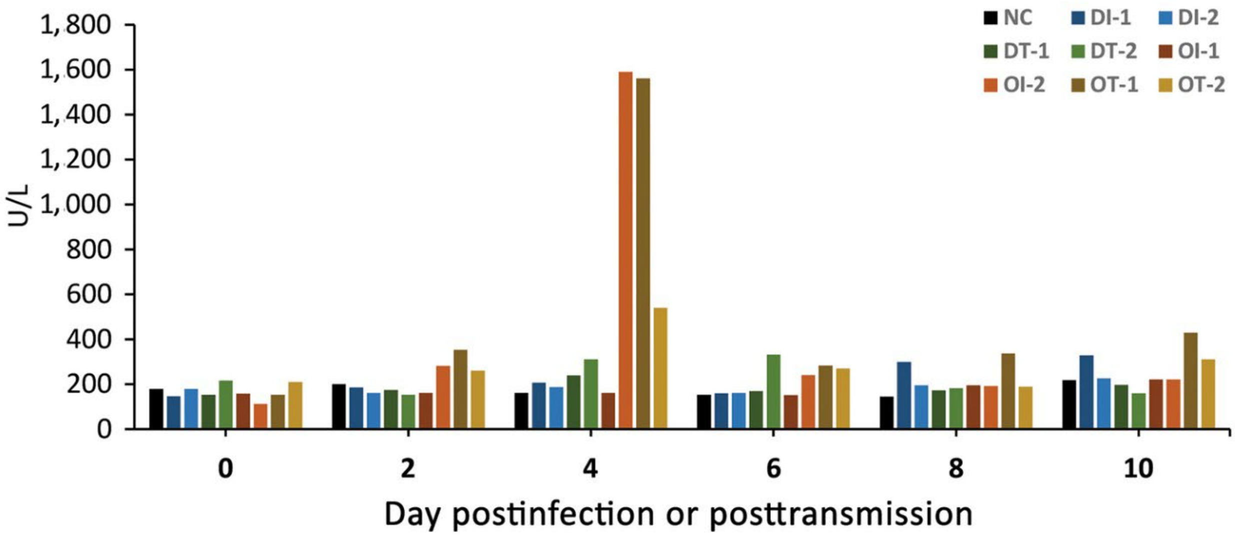

Appendix Figure. Creatine kinase levels in dogs infected with and or exposed to transmission of Delta or Omicron variants. We designated 1 negative control dog, 2 each infected with Delta or Omicron variant, and 2 each cohoused with infected dogs (i.e., transmission dogs). In the key, individual dogs are identified as -1 and -2 along with a status designation. NC, negative control; DT, Delta transmission dog; DI, Delta-infected dog; OT, Omicron transmission dog; OI, Omicron-infected dog
